# Tension Pneumomediastinum: A life-threatening condition in patients with COVID-19

**DOI:** 10.5339/qmj.2021.55

**Published:** 2021-10-19

**Authors:** Nissar Shaikh, Gamal Al Ameri, Muhsen Shaheen, Wael I. Abdaljawad, Sujith Prabhakaran, Mohammad Al Wraidat, Ahmed S. Mohmed, Mohamad Y. Khatib, Abdulqadir J. Nashwan

**Affiliations:** ^1^Surgical Intensive Care Department, Hamad General Hospital (HGH), Hamad Medical Corporation (HMC), Doha, Qatar E-mail: anashwan@hamad.qa; ^2^Critical Care Department, Hazm Mebaireek General Hospital (HMGH), Hamad Medical Corporation (HMC), Doha, Qatar; ^3^Medical Intensive Care Department, Hamad General Hospital (HGH), Hamad Medical Corporation (HMC), Doha, Qatar

**Keywords:** acute respiratory distress syndrome, COVID-19, chest drain, invasive ventilation, tension pneumomediastinum

## Abstract

Tension pneumomediastinum (TPM) is a rare but potentially fatal clinical entity. TPM leads to the leakage of air into the mediastinal cavity and increased pressure in thoracic vessels, respiratory tract, and the heart. Herein, this report presents a series of five cases of coronavirus disease-2019 (COVID-19) that caused acute respiratory distress syndrome (ARDS) and TPM. All patients were male who had severe ARDS with a secondary lung infection that required invasive ventilation and had moderate positive-end expiratory pressure. All patients required vasopressors to maintain hemodynamics, and two patients needed decompression with chest drains. One patient received extracorporeal membrane oxygenation therapy. Three patients had cardiac arrest, and two patients died; thus, the mortality rate was 40%. Patients with COVID-19 pneumonia with ARDS required invasive ventilation and prone positioning. Secondary lung infection can cause TPM, and TPM may cause cardiac arrest. Management should be prompt recognition and decompression with the insertion of drains, and conservative treatment is required in stable cases. Protocols for the management of pneumomediastinum and TPM may enable early detection, earlier management, and prevention of TPM.

## Background

Pneumomediastinum is a clinical entity defined as the presence of air in the mediastinum. However, in rare cases, excessive air in the mediastinal space can increase pressure on vital cardiovascular and respiratory systems, causing cardiovascular collapse. This phenomenon is known as tension pneumomediastinum (TPM). TPM is a potentially fatal clinical condition initially described in the 1940s and frequently reported in patients with pulmonary tuberculosis.^1^ Severe coronavirus disease 2019 (COVID-19) pneumonia may progress to full-blown acute respiratory distress syndrome (ARDS) within a short period.^2^ Thus, patients require invasive ventilation with high ventilator settings to maintain their oxygenation requirements. These patients are at a higher risk of developing pneumomediastinum and eventually TPM. The literature search revealed one case report of TPM and a case series titled “Pneumomediastinum in COVID-19 patients.”^3-4^ Herein, this report presents another case series of five patients with COVID-19 pneumonia who developed TPM while on invasive ventilation.

All patients were admitted to the tertiary COVID-19 care facility in Hamad Medical Corporation: Doha-Qatar. After obtaining permission from the local medical research center (MRC-04-1191) and consent from the patient or patient's relative, patients’ files were reviewed, and data were collected retrospectively.

## Case Series

### Case 1

A 51-year-old man, nonsmoker without a history of previous medical illness, presented in May 2020 with severe COVID-19 pneumonia. He had fever and myalgia for 3 days. He was brought by ambulance to the emergency department and was immediately admitted to the intensive care unit (ICU). He had shortness of breath and low oxygen saturation, requiring noninvasive ventilation in the initial period. His chest radiographs showed bilateral peripheral infiltrates. He was managed as per the hospital COVID-19 protocol at that time. Initially, he was advised self-prone positioning with oxygen supplementation, but on day 4, his condition deteriorated, requiring intubation and ventilation. During his ICU stay, he developed a cytokine storm requiring interleukin (IL-6) antagonist tocilizumab and steroids. He had a prolonged course of invasive ventilation during which he was positioned prone multiple times. He received convalescent plasma and was successfully extubated on day 15 after improvement in ventilatory settings. He remained stable for the next 4 days but developed severe sepsis, leading to septic shock and ARDS and eventually reintubation. Sputum cultures grew *Stenotrophomonas maltophilia* and *Klebsiella*, which was extended-spectrum beta-lactamase resistant.

**Figure 1.**
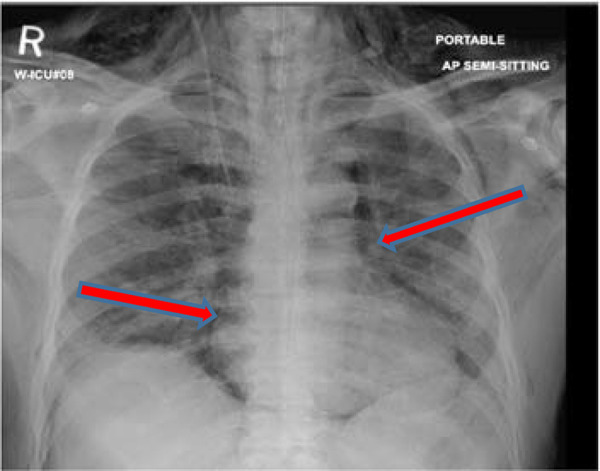
Chest X-ray image showing pneumomediastinum

Ventilator support continued for another 8 days, and on day 28 after his admission, he developed extensive lower neck and chest wall subcutaneous emphysema. Chest X-ray (CXR) examination showed pneumomediastinum (Figure 1). No evident pneumothorax was noted on the CXR on that day. During this period, he had been on high ventilatory setting, including controlled mandatory ventilation (CMV) with FiO_2_ of 50%, tidal volume of 350 ml, positive-end expiratory pressure (PEEP) of 8 mmHg, and respiratory rate of 30 breaths per minute.

**Figure 2.**
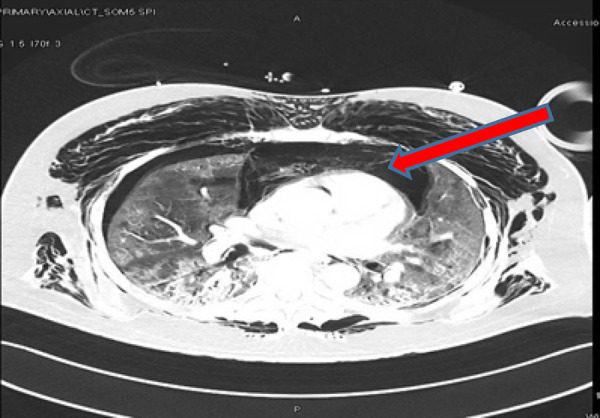
Computed tomography of the chest showing tension pneumomediastinum with pneumothorax

Computed tomography (CT) was performed, which showed extensive pneumomediastinum and bilateral pneumothorax. A small area of possible disintegration and discontinuation within the right posterolateral wall of the lower part of the trachea was noted (tracheal tear). Diffuse ground-glass opacities were observed in both lung fields, which were consistent with the underlying ARDS (Figure 2).

Chest drains were inserted bilaterally to relieve the tension pneumothorax. The patient's condition deteriorated in the next few days due to multiorgan failure, and he expired on day 36 in the ICU.

### Case 2

A 71-year-old man, nonsmoker with a past medical history of osteoarthritis and hypertension being managed with amlodipine, presented to the hospital with signs of COVID-19 pneumonia and moderate ARDS. He had cough, fever, and myalgia for 2 days. He was brought by ambulance and immediately admitted to the ICU via the emergency department. He was initially managed with a non-rebreathing oxygen mask, self-prone positioning, and noninvasive ventilation. He also received antibiotics, antivirals, and steroids. As he showed signs of cytokine storm, tocilizumab was given. Later, he also received convalescent plasma from recovered COVID-19 donors. The management was performed according to the hospital's COVID-19 management guidelines. During his hospitalization, he developed hospital-acquired secondary pneumonia due to *Enterobacter cloacae*, *Candida*, and *Acinetobacter baumanni*. Bronchoalveolar lavage grew *S. maltophilia*. He was intubated and ventilated on day 32 because of worsening ARDS, sepsis, and multiorgan dysfunction syndrome (MODS).

**Figure 3.**
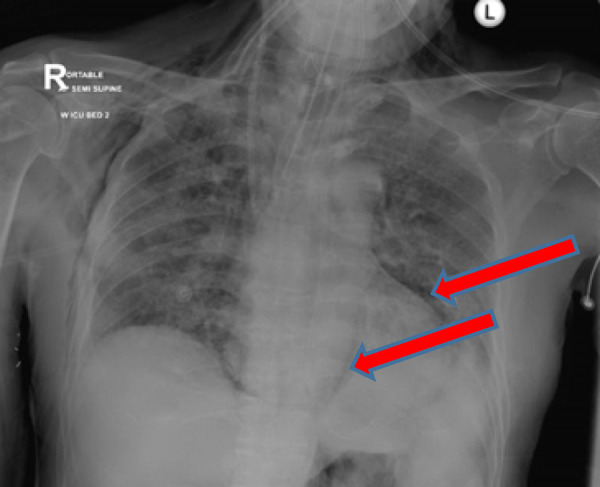
Chest X-ray image showing pneumomediastinum

Ventilatory requirements remained high during this period, and on day 37, his chest radiograph showed pneumomediastinum and subcutaneous emphysema with no pneumothorax (Figure 3). His ventilator settings on that day were CMV mode with FiO2 of 40% and PEEP of 8 mmHg, tidal volume of 370 ml, and respiratory rate of 26 breaths per minute.

**Figure 4.**
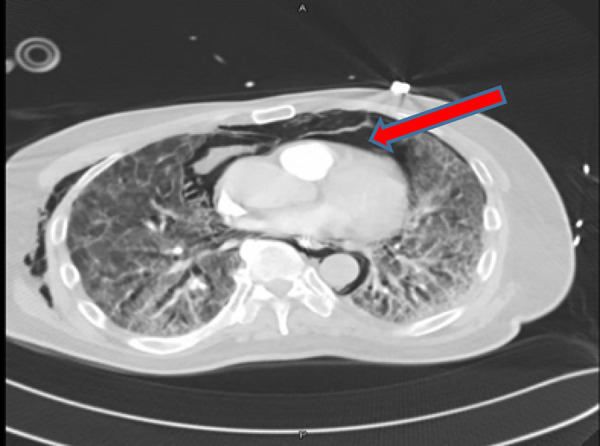
Computed tomography scan of the chest showing tension pneumomediastinum

CT showed extensive surgical emphysema in the neck and anterolateral aspect of the chest wall, which was more prominent on the right side. Extensive tension pneumomediastinum was noted extending from the superior mediastinum, anterior mediastinum, and posterior mediastinum and extending even to the retroperitoneum space surrounding the pancreas and left kidney (Figure 4). No evidence of pneumoperitoneum was found. A bilaterally thin rim of pneumothorax was noted slightly more on the left side. No evidence of lung collapse was seen in the CT scan.

Conservative management was adopted for his pneumomediastinum by reducing the ventilator pressures and close observation. The condition resolved over time. He was tracheostomized on day 12 after intubation due to a prolonged ventilatory course. He developed recurrent sepsis during his ICU stay; however, timely management improved his condition over time. The patient was discharged from the ICU on day 29 after intubation.

### Case 3

**Figure 5.**
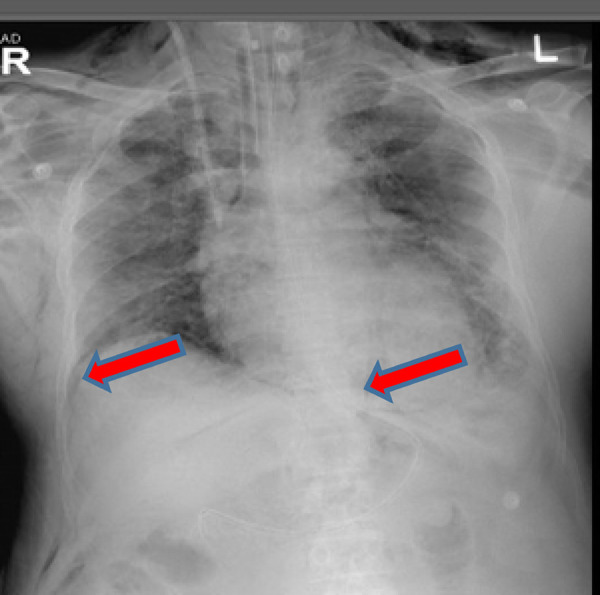
Chest X-ray image showing pneumomediastinum

A 58-year-old man, an ex-smoker, was diagnosed with COVID-19 pneumonia. He had a complicated past medical history, including diabetes mellitus, end-stage renal disease, post-renal transplant status, and pulmonary tuberculosis 3 years ago. He had completed a full course of anti-tuberculosis therapy. He presented with worsening shortness of breath and severe pneumonia, requiring ICU admission from a COVID-19 facility (2 days), and he was intubated on day 3. During his ICU stay, he developed severe sepsis and MODS, requiring antibiotics, antivirals, and steroids. He also showed signs of cytokine storm, so tocilizumab was administered. Later, he also received convalescent plasma. All management was performed according to the hospital's COVID-19 management protocol. Prone positioning was performed several times during his ICU stay. His tracheal aspirate culture grew *Aspergillus ochraceus* and *Candida tropicalis*. On day 22, CXR imaging showed subcutaneous emphysema with no pneumothorax but was suspicious of pneumomediastinum (Figure 5). His ventilatory settings were CMV with FiO2 of 40%, PEEP of 10 mmHg, tidal volume of 4–6 ml/kg, and a high respiratory rate (28 breaths per minute).

**Figure 6.**
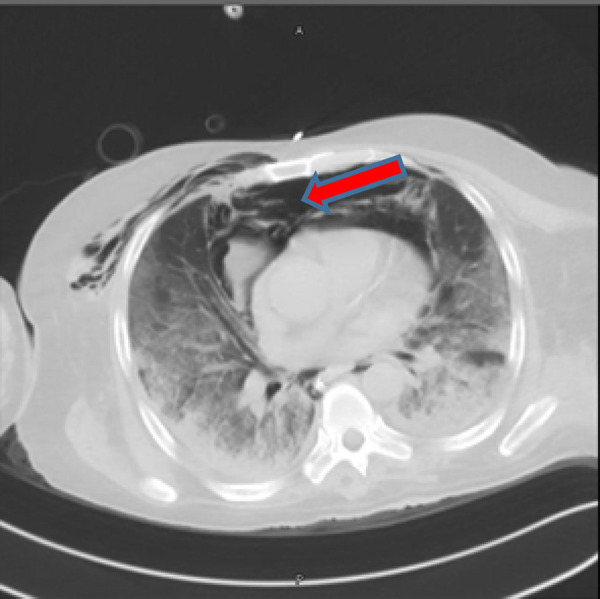
Computed tomography of the chest showing tension pneumomediastinum

CT of the chest revealed a significant amount of air within the mediastinum (TPM). Anterior chest wall emphysema was also noted. There was no evidence of pleural effusion or pneumothorax (Figure 6). Bilateral diffuse ground-glass appearance and airspace opacities involving all lung lobes were seen.

The pneumomediastinum was managed conservatively and was resolved without any intervention at that time. His condition deteriorated further during his ICU stay because of severe sepsis and ARDS. Vasopressor requirements increased, and he suffered a cardiac arrest on day 32. The cardiac arrest was reverted, but due to severe brain insult, his condition did not improve. He was transferred from the ICU for long-term care and rehabilitation.

### Case 4

**Figure 7.**
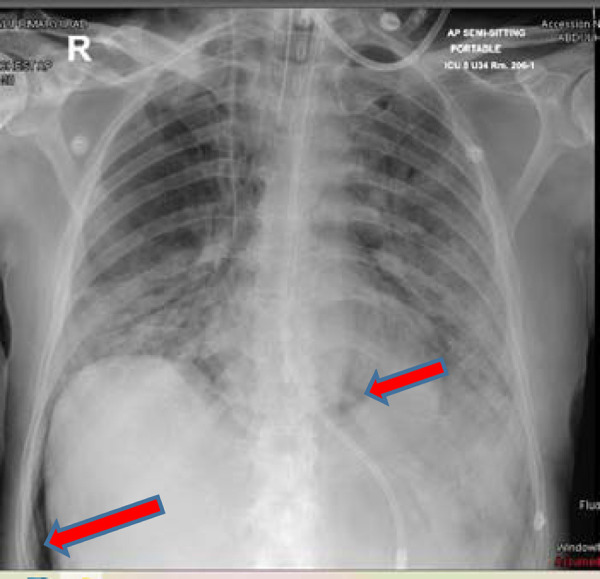
Chest X-ray image showing pneumomediastinum and pneumoperitoneum

A 63-year-old man, a nonsmoker, with a past medical history of type 2 diabetes and diabetic nephropathy presented with sudden deterioration of cognitive function, generalized fatigability, and hematuria. He was diagnosed with COVID-19 and directly transferred to the ICU from a peripheral COVID-19 facility. The patient stayed in a COVID-19 facility for 3 days. His condition deteriorated, and he developed COVID-19 pneumonia and ARDS requiring intubation on day 18 of admission. He was managed with antivirals and antibiotics, as he also showed signs of cytokine storm requiring tocilizumab and steroids. At 10 days after intubation, his CXR showed signs of pneumoperitoneum and anterior mediastinal air (Figure 7). The ventilator settings included pressure control mode with FiO_2_ of 60%, PEEP of 8 mmHg, pressure control of 32, and respiratory rate of 28 breaths per min. His sputum culture was growing *Klebsiella*, *Candida albicans*, and *Serratia marcescens*.

**Figure 8.**
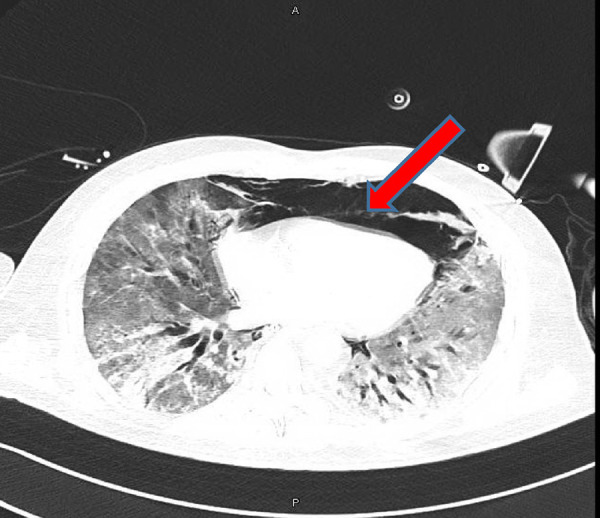
Computed tomography scan showing tension pneumomediastinum

CT showed mild lower neck surgical emphysema predominant in the right lateral side. Extensive TPM extended from the superior mediastinum and down anteriorly and posteriorly to the pre-crural and precardiac space causing cardiac tension. This pneumomediastinum extended inferiorly to the anterior retroperitoneal space. No intraperitoneal free air was seen. Diffuse mosaic ground-glass attenuation of both lungs was noted, which was consistent with ARDS (Figure 8).

Pneumomediastinum was managed conservatively, and the patient recovered from this. Later, he developed severe sepsis, multiorgan dysfunction, and lung fibrosis and unfortunately expired 1 month later.

### Case 5

**Figure 9.**
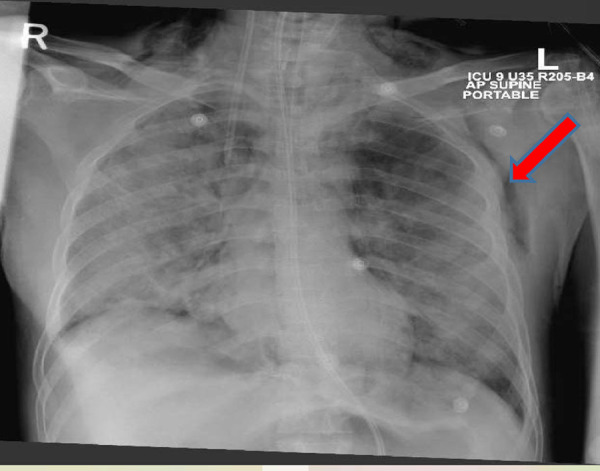
Chest X-ray image showing subcutaneous emphysema

A 51-year-old man, a nonsmoker, with a past medical history of type 2 diabetes was initially admitted to the peripheral COVID-19 facility for 3 days. However, he had worsening COVID-19 pneumonia and ARDS, which prompted his transfer to the ICU and intubation on day 10. He developed sepsis and multiorgan dysfunction. He was managed with antivirals, antibiotics, tocilizumab, and steroids. Two days after intubation, his CXR showed left-sided subcutaneous emphysema and signs of pneumomediastinum (Figure 9). His ventilator settings were at CMV mode with a tidal volume of 390 ml, PEEP of 8 mmHg, and FiO_2_ of 50%. During his ICU stay, he experienced severe sepsis and MODS that required antibiotics, antivirals, and steroids. He also showed signs of cytokine storm requiring tocilizumab.

**Figure 10.**
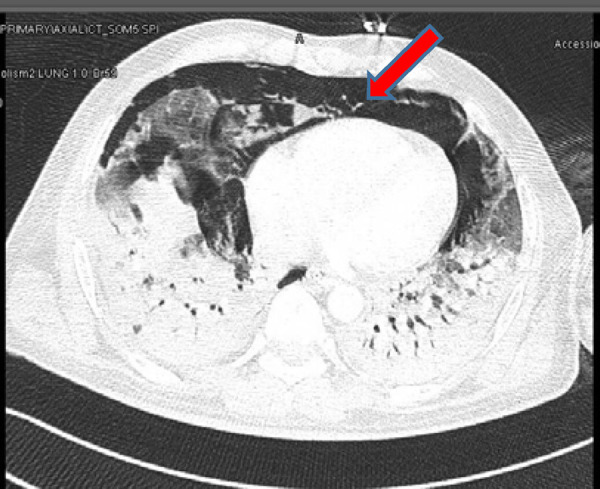
Computed tomography of the chest showing tension pneumomediastinum

CT demonstrated extensive pneumomediastinum and subcutaneous emphysema with mild pneumothoraces bilaterally. The surgical emphysema extended into the neck. The pneumomediastinum extended into the abdomen and appeared anteriorly in the extraperitoneal region (Figure 10). Bilateral segmental branches of the main pulmonary arteries, going toward the lower lobes, showed filling defects.

The patient also had significant pneumothorax; thus, a right-sided chest drain tube was inserted. A few hours later, he had cardiac arrest due to refractory hypoxia requiring ECMO support. He responded to the management and was decannulated from ECMO after 29 days. His sputum grew *Enterococcus fecalis*, *Klebsiella*, and *S. marcescens*. He recovered after a prolonged ICU stay and was discharged for rehabilitation.

Key details of all five cases are presented in Table 1.

## Discussion

TPM is caused by direct injury or barotrauma to the tracheobronchial tree, alveoli, or esophagus, leading to air leakage into the mediastinum and causing tension in the closed cavity. This may cause compression of large vessels, heart, and lungs leading to cardiovascular and respiratory compromise, which can threaten the patient's life.^1-4^ COVID-19 initially affects the respiratory system causing pneumonia. Complications include ARDS requiring invasive ventilation with higher or maximum ventilator settings. This situation carries an increased risk of barotrauma to the lungs and tracheobronchial tree. However, there is not much literature about COVID-19 pneumonia that complicates into TPM or pneumomediastinum; there is only one case report of TPM, and a series of eight cases of pneumomediastinum are described to date.^2,3^ In our institution, a total of 1100 patients with COVID-19 were admitted, 3.9% had spontaneous pneumothorax and pneumomediastinum, and 5 (0.45%) had TPM.

COVID-19 is known to cause airway inflammation and edema, which put these patients at a higher risk of airway tract injuries following instrumentation.^3^ Placement of a large-sized endotracheal tube also carries a risk of TPM development in patients with COVID-19.^3^ In this case series, only one patient had a tracheal injury, which may have contributed to the TPM, and the trauma literature describes that pneumomediastinum and pneumothorax caused by tracheal injury account for 10% of the cases. This tracheal injury causes the leakage of air into neck spaces, and with continuous air leakage and positive pressure ventilation, air may spread into the mediastinal space.^6^ Three patients required reintubation because of secondary bacterial and fungal pneumonia, and ARDS, in combination with primary COVID-19 pneumonia with alveolar membrane destruction, may increase the risk of TPM.

All our patients received intermittent prone positioning intervention after the intubation as a therapeutic approach for respiratory failure due to COVID-19. Prone positioning in patients with acute respiratory distress is a known risk factor for pneumomediastinum and TPM.^7^ Wali et al. described in their case series that one of their patients with COVID-19 developed pneumomediastinum immediately after prone positioning.^3^ All our patients had pneumo-mediastinum at day 10 post-intubation or later. They had secondary bacterial and/or fungal pulmonary infections. The majority of our patients also had complex previous medical histories, which may contribute to their frail condition.

For the diagnosis of TPM apart from hemodynamic instability, imaging studies are confirmatory. The initial CXR will show the presence of air in the mediastinum and around or earth-heart sign due to the collapsed and restricted filling of the heart chambers.^8^ Sometimes, it is difficult to see the heart shadow in the X-ray image of patients with TPM; hence, it is called a “vanished heart sign.” This sign is the disappearance of the heart shadow due to air around the heart in the mediastinum.^9^ In a few patients with TPM, the “earth-heart sign” observed in the CXR and CT image reflects cardiac compression due to TPM. The cardiac shadow appears to be flattened. This is called the earth-heart sign because the heart shape resembles the shape of an oblate sphere as the earth.^10^ A chest CT can show a more detailed extension of air in the mediastinum, including the retromediastinum.^3^

TPM is treated with the insertion of drains through the suprasternum or xiphisternum to decompress the mediastinum. The conservative management includes reducing airway pressures, allowing permissive hypercapnia and denitrogenation of the mediastinum air by increasing the percentage of oxygen supplementation.^2,3^ Two of our patients had intercostal drains, and three patients received conservative management. One patient required extracorporeal membrane oxygenation (ECMO) therapy. Hassan et al. described the successful use of ECMO in patients with ARDS due to Middle East respiratory syndrome outbreak in the Arabian Peninsula.^11^ One of the conservatively managed patients and one patient managed with drainage of TPM died later because of other complications. Quincho-Lopez^12^ described two cases of COVID-9 pneumonia complicated with pneumomediastinum. Shan et al.^13^ described a COVID-19 case complicated by pneumomediastinum; all these patients had better outcomes and discharged from the hospital. Pooni et al described a case of mediastinitis complicated by pneumomediastinum.^14^ More recently,Agrawal et al. described four cases of pneumomediastinum in patients with COVID-19 with two fatalities.^15^ Machiraju et al. described three cases of pneumomediastinum with two case fatalities.^16^ All these studies mentioned pneumomediastinum only, whereas our case series describes all patients with TPM and hence a few patients had not recovered and died.

## Conclusion

Patients with COVID-19 pneumonia and ARDS developing secondary infection have an increased risk for tension pneumomediastinum. Patients with COVID-19 pneumonia with ARDS requiring invasive ventilation and prone positioning may lead to TPM. TPM can cause cardiac arrest. Early recognition and management are vital to prevent complications. Management should be prompt decompression with drain insertion or conservative management in stable cases. ECMO is useful in patients with TPM who are not responding to conventional invasive ventilation. Protocols for the management of pneumomediastinum and TPM may detect the disease early, with earlier management and prevention of TPM.

## Declarations

### Ethics approval and consent to participate

The article describes a case series. Therefore, no additional permission from our Ethics Committee was required (MRC-04-20-1191).

### Consent for publication

The consents for publication were obtained from all included patients.

### Availability of data and material

All data generated or analyzed during this study are included in this published article.

### Competing interests

The authors declare that they have no competing interests.

### Funding

This study was not funded.

### Authors’ contributions

Data Collection: GAA and MAW

Literature Search: NSH, MSH, WIA, and SPR

Manuscript Preparation: NSH, AJN, ASM, and MKH.

All authors read and approved the final manuscript.

### Acknowledgments

None.

## Figures and Tables

**Table 1:** Clinical characteristics and outcomes of the five cases

Patient	Gender	Age	Signs and symptoms	PEEP/Plateau	Tracheal injury	Hemodynamics	Cardiac arrest	Chest drains	ECMO	Outcome

1	M	51	Severe ARDS P/F 90	8/28	Yes	Vasopressors	No	Yes	No	Died

2	M	71	Moderate ARDS P/F 120	8/30	No	Vasopressors	No	No	No	Survived

3	M	58	Severe ARDS P/F 70	10/30	No	Vasopressors	Yes	No	No	Hypoxic brain/survived

4	M	63	Severe ARDS P/F 60	8/29	No	Vasopressors	Yes	No	No	Died

5	M	51	Severe ARDS P/F 60	8/30	No	Vasopressors	Yes	Yes	Yes	Survived


ARDS, acute respiratory disease syndrome
